# Oligodendrocyte and Interneuron Density in Hippocampal Subfields in Schizophrenia and Association of Oligodendrocyte Number with Cognitive Deficits

**DOI:** 10.3389/fncel.2016.00078

**Published:** 2016-03-30

**Authors:** Peter Falkai, Johann Steiner, Berend Malchow, Jawid Shariati, Andreas Knaus, Hans-Gert Bernstein, Thomas Schneider-Axmann, Theo Kraus, Alkomiet Hasan, Bernhard Bogerts, Andrea Schmitt

**Affiliations:** ^1^Department of Psychiatry and Psychotherapy, Ludwig Maximilians-University MunichMunich, Germany; ^2^Department of Psychiatry and Psychotherapy, University of MagdeburgMagdeburg, Germany; ^3^Department of Psychiatry and Psychotherapy, University of GöttingenGöttingen, Germany; ^4^Center for Neuropathology and Prion Research (ZNP), Ludwig Maximilians-University MunichMunich, Germany; ^5^Laboratory of Neuroscience (LIM27), Institute of Psychiatry, University of São PauloSão Paulo, Brazil

**Keywords:** schizophrenia, hippocampus, oligodendrocytes, interneurons, cognition, immunohistochemistry, post-mortem

## Abstract

In schizophrenia, previous stereological post-mortem investigations of anterior, posterior, and total hippocampal subfields showed no alterations in total neuron number but did show decreased oligodendrocyte numbers in CA4, an area that corresponds to the polymorph layer of the dentate gyrus (DG). However, these investigations identified oligodendrocytes only on the basis of morphological criteria in Nissl staining and did not assess alterations of interneurons with immunohistochemical markers. Moreover, the association of findings in the posterior hippocampus with cognitive deficits remains unknown. On the basis of the available clinical records, we compared patients with definite and possible cognitive dysfunction; nine patients had evidence in their records of either definite (*n* = 4) or possible (*n* = 5) cognitive dysfunction. Additionally, we assessed the density of two oligodendrocyte subpopulations immunostained by the oligodendrocyte transcription factors Olig1 and Olig2 and of interneurons immunolabeled by parvalbumin. We investigated posterior hippocampal subregions in the post-mortem brains of the same schizophrenia patients (SZ; *n* = 10) and healthy controls (*n* = 10) we examined in our previously published stereological studies. Our stereological studies found that patients with definite cognitive deficits had decreased total/Nissl-stained oligodendrocyte numbers in the left (*p* = 0.014) and right (*p* = 0.050) CA4, left CA2/3 (*p* = 0.050), left CA1 (*p* = 0.027), and left (*p* = 0.050) and right (*p* = 0.014) subiculum of the anterior part of the hippocampus compared to patients with possible cognitive deficits. In the present study, we found no significant influence of definite cognitive deficits in the posterior part of the hippocampus, whereas in the entire hippocampus SZ with definite cognitive deficits showed decreased oligodendrocyte numbers in the left (*p* = 0.050) and right (*p* = 0.050) DG and left CA2/3 (*p* = 0.050). We did not find significant differences in Olig1-, Olig2-, or parvalbumin-positive cell density between SZ and controls in any of the subregions of the posterior hippocampus. Based on the results from our stereological study we hypothesize that a decreased number of oligodendrocytes in the anterior and entire hippocampus may be involved in cognitive deficits by impairing the connectivity of this structure in schizophrenia. In the posterior hippocampus, we could not replicate previously reported findings of decreased interneurons from the entire hippocampus.

## Introduction

Schizophrenia has an unfavorable outcome in more than half of all patients and is linked to social and vocational disability (an der Heiden and Häfner, 2011). This unfavorable outcome is mainly due to negative symptoms, including cognitive dysfunction. While positive symptoms can be treated well by antipsychotics when given according to current guidelines (Hasan et al., 2012), effective treatment options for negative symptoms are still lacking. In this context, a recent meta-analysis revealed that cognitive dysfunction and residual negative symptoms are widely resistant to any known treatment (Fusar-Poli et al., 2015). Another meta-analysis, however, showed that antipsychotics have at least a moderate effect on cognitive impairments in schizophrenia (Desamericq et al., 2014). Cognitive impairments in schizophrenia involve deficits in episodic and working memory, for example, as well as in attention, processing speed, and problem solving (Nuechterlein et al., 2004; Falkai et al., 2011).

A cognitive cluster representing diminished verbal memory is related to decreased volume of the hippocampus (Geisler et al., 2015). Interestingly, in schizophrenia these verbal memory deficits have been found to be correlated with a loss of hippocampal volume, especially on the left side (Hasan et al., 2014). Recent examination of structural magnetic resonance imaging-based alterations in hippocampal subfields revealed decreased volumes of the cornu ammonis (CA) CA4/dentate gyrus (DG), CA2/3, and subiculum in schizophrenia patients (SZ) compared to healthy controls (Haukvik et al., 2015), but underlying alterations on the cellular level and their relationship to cognitive deficits remain unknown.

Recently, our group investigated the cytoarchitecture of the posterior, anterior, and entire hippocampus in post-mortem schizophrenia brains by evaluating neuronal, oligodendrocyte, and astrocyte numbers with design-based stereological estimation. In the posterior area, we found a significant reduction of oligodendrocyte numbers in the left and right CA4 regions (Schmitt et al., 2009). In the anterior part, we found a decreased number of oligodendrocytes in the left CA4, fewer neurons in the left DG, and smaller volumes of both the left CA4 and DG in SZ compared to healthy controls. In the entire hippocampus, both decreased oligodendrocyte numbers in the left CA4 and reduced volume remained significant (Falkai et al., 2016). In the Nissl-stained brain sections, however, we identified oligodendrocytes on the basis of morphological criteria only and did not distinguish between pyramidal neurons and interneurons. This approach may be relevant to our results, because in a stereological study, somatostatin-stained interneurons have been reported to be reduced in CA4, CA2/3, and CA1 and parvalbumin-stained interneurons in CA4 and CA1, although the total number of neurons was unchanged (Konradi et al., 2011). Moreover, the clinical consequence of our findings and the involvement of a deficit of premature or mature oligodendrocytes remains unknown. Oligodendrocytes are involved in myelination and ensure proper connectivity in neuronal networks. In SZ, deficits in myelination and brain connectivity have been found (Cassoli et al., 2015; Saia-Cereda et al., 2015), which are hypothesized to be related to symptoms of the disease and cognitive deficits (Mitterauer, 2011). In fact, in diffusion tensor imaging (DTI) studies verbal memory has been reported to be associated with decreased left-side dominant fractional anisotropy in the hippocampus and fornix of SZ (Lim et al., 2006; Nestor et al., 2007).

The aim of the present study was to address the question whether the findings of this study and those of our previous stereological studies in the anterior, posterior, and entire hippocampus (Schmitt et al., 2009; Falkai et al., 2016) can be connected to simple measures of cognitive dysfunction in this illness. Furthermore, we investigated whether the density of more premature oligodendrocytes, stained by the oligodendrocyte transcription factors Olig1, or of more mature oligodendrocytes stained by Olig2 respectively, or of parvalbumin-positive interneurons is altered in the posterior part of the hippocampus in the same post-mortem schizophrenia brains as previously investigated by design-based stereology in histologically stained sections (Schmitt et al., 2009; Falkai et al., 2016).

## Materials and Methods

### Participant Characteristics

Post-mortem brains were obtained from the Düsseldorf Brain Collection (Bogerts et al., 1990) and the use of post-mortem material has been approved by the Ethics Committee of University of Magdeburg. Patients fulfilled ICD-9 criteria for schizophrenia and had been treated with typical antipsychotics for most of their illness. Exclusion criteria were alcohol or drug abuse and other neuropsychiatric disorders. We investigated the same sample as in our previously published stereological study of the posterior hippocampus in schizophrenia (Schmitt et al., 2009). Briefly, we assessed 10 patients with schizophrenia (mean [SD] age 55.1 [7.7] years; five males, five females; mean [SD] post-mortem interval [PMI] 42.0 [17.2] h; mean [SD] disease duration: 23.0 [4.9] years) and 10 age- and gender-matched healthy controls without a history of a neuropsychiatric disorder, alcohol or drug abuse, dementia, neurological illness, trauma, or chronic terminal disease (mean [SD] age 50.2 [10.1] years; 5 males, 5 females; mean [SD] PMI 36.8 [20.3] h; Table 1). The mean daily dose of neuroleptic treatment during the last 90 days had been documented and calculated in chlorpromazine equivalents (CPE; Rey et al., 1989). Brains were uniformly fixed in toto in 10% phosphate buffered paraformaldehyde for about 7 months (pH 7.0, *t* = 15–20°C). Then, the frontal and occipital parts of the brain were separated from the middle part, which was located between the lateral geniculate nucleus and the splenium of the corpus callosum, i.e., containing the anterior and posterior hippocampus. From this middle part, 20 μm coronal sections containing both hemispheres were cut on a Polycut S Leica microtome. The thickness of each section was determined by focusing through the section and subtracting the z-axis coordinate of the lower from that of the upper surface by the microcator, part of the Olympus microscope. The mean (SD) section thickness after histological procedure was 18.7 (1.1) μm.

**Table 1:** **Characteristics of healthy controls and schizophrenia patients (SZ) and healthy controls investigated in the present study of the posterior hippocampus**.

**Schizophrenia patients**
No.	S	A	D	C	CPE	Cause of death	Last neuroleptic treatment
P3	M	48	18	2	3500	Cardiac failure	Haloperidol, Haldoperidol Decanoate, Zuclopenthixol
P9	M	65	26	1	540	Pulmonary insufficiency	Haloperidol, Fluphenazine
P10	M	46	18	1	250	Pulmonary embolism	Clozapine, Levomepromazine
P14	M	51	28	2	350	Ileus	Perphenazine, Promethazine
P20	M	47	23	2	640	Cardiac arrest	Fluphenazine, Benperidol, Levomepromazine
P7	F	66	30	1	800	Myocardial infarction	Benperidole, Fluphenazine
P12	F	53	20	2	2495	Myocardial infarction	Perphenazine, Pericyazine
P13	F	60	16	1	0	Pneumonia	Fluphenazine
P18	F	63	23	1	988	Suicide	Haloperidol
P23	F	52	28	n.a.	n.a.	Suicide by drowning	n.a.
**Healthy controls**
N29	M	47				Coronary thrombosis	
N20	M	64				Aneurysm rupture	
N3	M	56				Pancreatitis	
N10	M	50				Myocardial infarction	
N23	M	38				Myocardial infarction	
N34	F	64				Peritonitis	
N22	F	47				Renal failure	
N27	F	50				Aneurysm rupture	
N21	F	52				Ovarial carcinoma	
N19	F	33				Coronary thrombosis	

*S = Gender (M = male, F = female), A = age at death (years), D = duration of disease, C = Cognitive deficits: 1 = possible cognitive deficits, 2 = definite cognitive deficits, CPE = dose of neuroleptics (mg/day) in chlorpromazine equivalents, n.a.= no data available*.

Within the hippocampus, we analyzed two sections per brain, taken from the posterior part of the hippocampal formation, which spanned from the lateral geniculate nucleus to the level of the splenium of the corpus callosum. The subregions to be investigated separately were the CA1, CA2/3, and CA4 (deep polymorph layer of the DG), and subiculum. CA2 and CA3 were lumped together as described in the literature because these small regions are difficult to separate at the microscopic level (Schmitt et al., 2009).

### Immunohistochemistry

Immunohistochemical staining was prepared as described previously (Mosebach et al., 2013). Briefly, sections were deparaffinized, and antigen demasking was performed by boiling the sections for 4 min in 10 mM citrate buffer (pH 6.0). Sections were then preincubated with 1.5% H_2_O_2_ for 10 min to block endogenous peroxidase activity. Non-specific binding sites were blocked with 10% normal goat serum for 60 min and washings with Phosphate buffered saline (PBS) buffer. Monoclonal mouse anti-Olig1 antibody (R&D Systems, Abingdon, UK, dilution 1:50) or rabbit anti-Olig2 antibody (Millipore/Merck Darmstadt, Germany, dilution 1:150) was applied for 72 h at 4°C for immunostaining of Olig1- or Olig2-positive oligodendrocytes, respectively. Then, the streptavidin-biotin technique was used to incubate sections with a biotinylated anti-mouse (GE Healthcare, Freiburg, Germany, dilution 1:100) or anti-rabbit (DakoCyomation, Glostrup, Denmark, dilution: 1:100) antibody, respectively. Chromogen 3,3’-diaminobenzidine (DAB) and ammonium nickel sulfate were used to visualize the reaction product. Parvalbumin-positive interneurons were immunolabeled under the same conditions with a monoclonal antibody to the protein (Sigma, Taufkirchen, Germany; dilution 1:500) and an anti-mouse peroxidase secondary antibody (Biozol, Germany, dilution 1:50; Bernstein et al., 2007).

### Evaluation of Olig1-, Olig2- and Parvalbumin-Stained Sections

**Figure 1:**
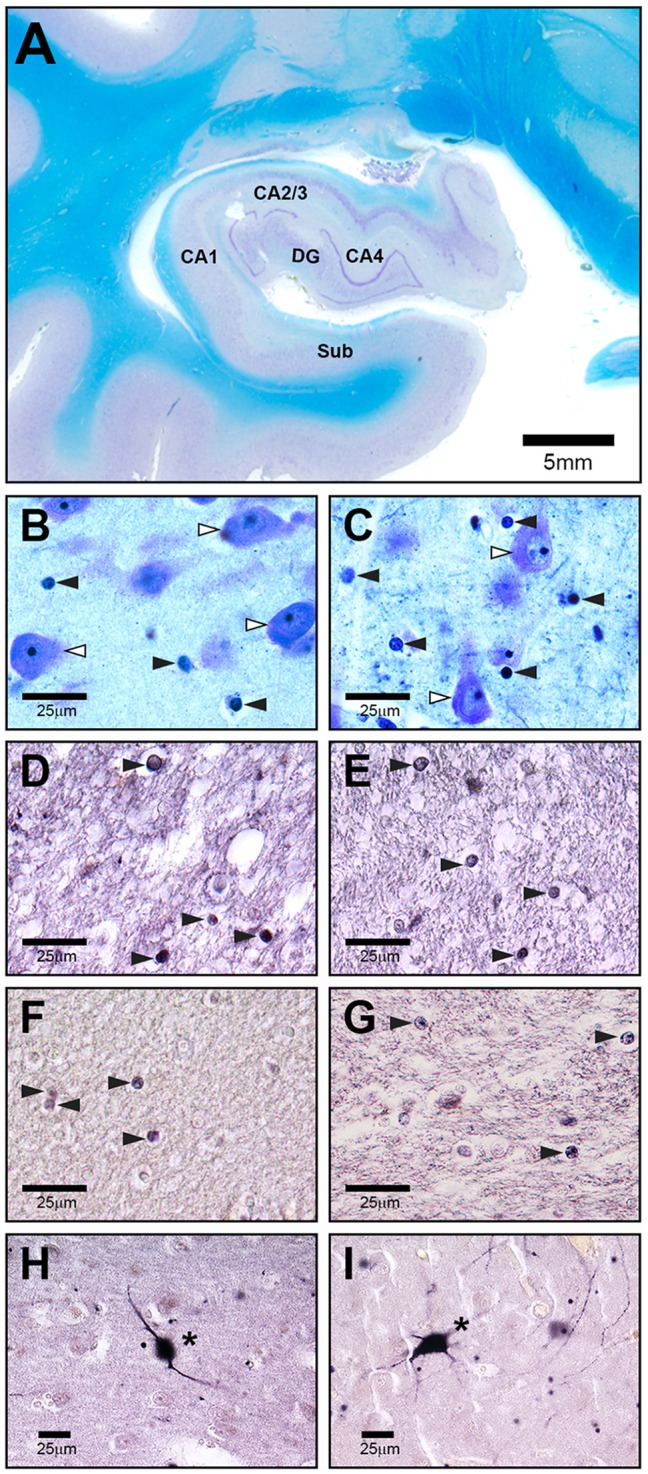
**Representative high-power photomicrographs of 20 μm-thick coronal sections from a schizophrenia patient (SZ) (C,E,G,I) and a healthy control (A,B,D,F,H). (A)** Overview of subregions including cornu ammonis (CA) 1, 2–3, 4, dentate gyrus (DG) and subiculum (Sub) in a Nissl and myelin stained section. The following sections show CA4. **(B,C)** White arrowheads indicate neurons, black arrowheads point to oligodendrocytes. **(D,E)** Olig1 immunostained stained section with black arrowheads showing oligodendrocytes. **(F,G)** Olig2 immunolabeled section with black arrowheads showing oligodendrocytes. **(H,I)** Parvalbumin stained section with asterisks pointing to immunopositive neurons.

A rater blinded to diagnosis analyzed the regions of interest in both hemispheres at 400× magnification by using a stereological workstation consisting of a modified light microscope (BX50; Olympus, Tokyo, Japan), Olympus Uplan Apo objectives (1.5×, 20×, 50×, 100× oil), motorized specimen stage for automatic sampling, electronic microcator, CCD color video camera, PC with frame grabber board, stereology software (Stereoinvestigator, MBF Bioscience Williston, VT, USA), and 17” monitor. Boundaries of hippocampal subfields were traced on video images displayed on the computer screen, and volumes were calculated in both sections. The numerical cell density of Olig1-, Olig2-, and parvalbumin-stained cells (Figure 1) was measured in the subregions by using the optional dissector and expressed as cells/mm^3^. Because the actual mean (SD) thickness of the sections was 18.7 (1.1) μm, two well-defined optical planes within the sections were used (distance 16 μm between the upper and lower guard zones), and all stained cells that came into focus while passing from the upper to the lower optical plane (plane of the dissector) were counted. The individual volume shrinkage factors (VSF) were calculated from the measured linear shrinkage factor (LSF) with the following formula: VSF = (LSF) (Mosebach et al., 2013). The intra-class correlation coefficients for intra-rater reliability were 0.81 for Olig1, 0.95 for Olig2, and 0.83 for the parvalbumin-stained cells.

### Evaluation of Cognitive Deficits in Schizophrenia Patients (SZ)

In our correlation analysis, we used results from Nissl-stained serial section of the anterior and posterior part of the hippocampus (Schmitt et al., 2009; Falkai et al., 2016). We searched for a surrogate marker of cognitive dysfunction in the group of SZ. Therefore, an independent psychiatrist (J. Steiner) who was blind to the histological results evaluated the medical records and used a three-point semi-quantitative scale to rate patients’ level of cognitive deficits (0 = no cognitive deficits, 1 = possible cognitive deficits, 2 = definite cognitive deficits). The rater searched for descriptions of deficits in the domains of attention, verbal learning, working memory, and executive functions and for indications of a decrease in cognitive performance or increase in residual symptoms over time. Patients who clearly had any of these symptoms were defined as having definite cognitive deficits; and those with marginal or borderline symptoms, possible cognitive deficits. Nine patients had either definite (*n* = 4) or possible (*n* = 5) deficits (Table 1); no records were available for one patient. It was not possible to formalize this kind of classification by scales (Ortakov et al., 1999).

### Statistical Analysis

The significance level was *α* = 0.05, and all tests were two-tailed. Statistical analyses were performed with IBM SPSS statistics 22.

In the first analysis, outcome measures were densities from immunohistochemical Olig1, Olig2, and parvalbumin stainings in the hippocampal subfields CA1, CA2/3, CA4, and the subiculum. The independent factor was diagnostic group (SZ, controls). Because Levene’s test detected significant variance inhomogeneities between the groups, we used non-parametric Mann-Whitney U tests to analyze diagnostic group differences. Cell densities were correlated with age at death, PMI, dose of neuroleptic treatment in CPE and disease duration by means of Spearman correlations for the total sample and separately for SZ and controls.

In addition to densities of oligodendrocytes and interneurons, outcome measures of the second analysis were the number of oligodendrocytes and neurons, estimated stereologically, and the structure volumes in the subfields (CA1, CA2/3, CA4, DG, and subiculum) of the anterior, posterior, and entire hippocampus of SZ, as published in Falkai et al. (2016). The independent factor was cognitive deficits (definite or possible). Means, standard deviations, and standard errors of the mean were calculated for all outcome measures for left and right hemispheres separately. We applied Levene’s test of variance homogeneity to compare the two groups with different degrees of cognitive deficits. Because this test found significant variance inhomogeneities for several variables, patients with definite and possible deficits were compared with non-parametric Mann-Whitney U tests.

For this explorative study with small group sizes, especially as regards the analysis of the influence of cognitive deficits on cell numbers or densities within the group of SZ, the results are presented without error probability correction. If a Bonferroni adjustment of the type I error probability had been applied, no significant differences would remain between SZ and controls. However, if the error probability was adjusted the power of detecting existing mean differences would be too low.

## Results

### Impact of Cognitive Deficits on Total Oligodendrocyte Numbers (Nissl Staining)

**Figure 2:**
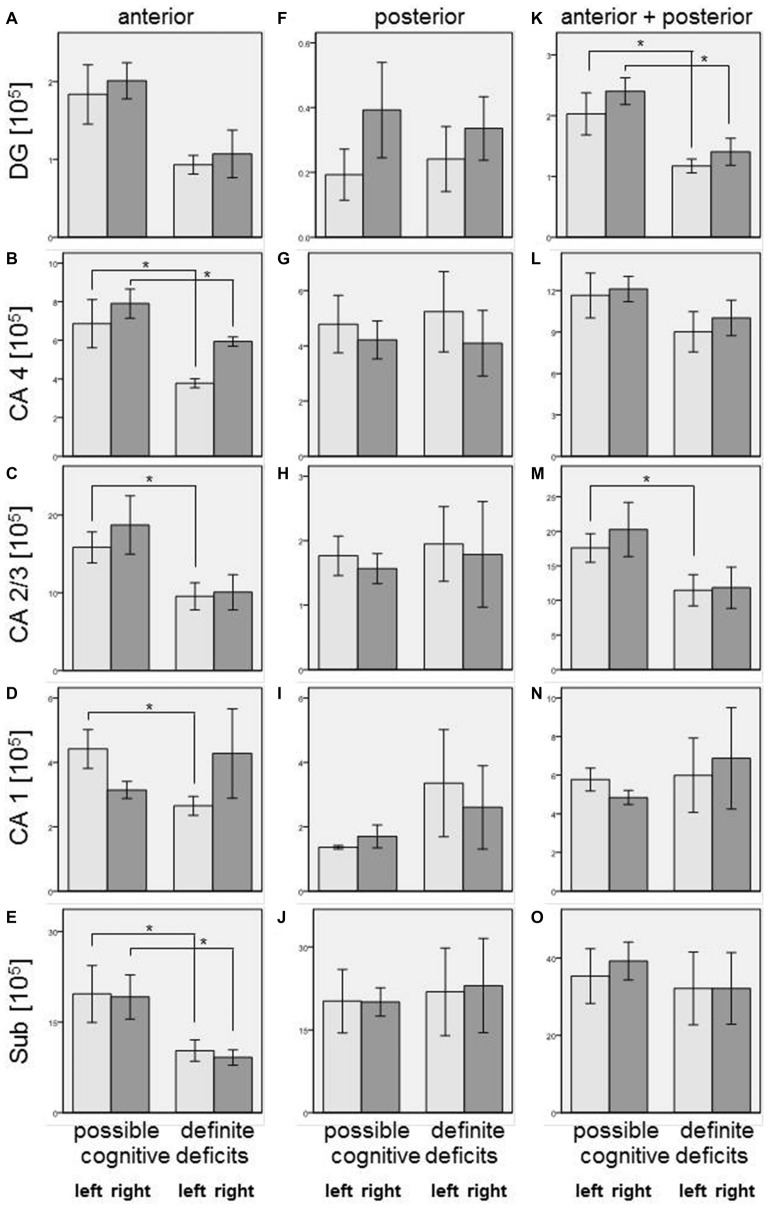
**Left column (A–E): Number of oligodendrocytes [×10^5^] in SZ with definite and possible cognitive deficits in anterior hippocampus: DG (DG, (A)), CA4 (B), CA2/3 (C), CA1 (D), and subiculum (Sub, (E)).** Columns represent mean oligodendrocyte number, and bars represent standard error of the mean. Light gray columns: left hemisphere; dark gray columns: right hemisphere. Decreased oligodendrocyte number in SZ with definite cognitive deficits were observed in CA4 (left and right), CA2/3 (left), CA1 (left), and subiculum (left and right). Middle column **(F–J)**: Number of oligodendrocytes [×10^5^] in SZ with definite and possible cognitive deficits in posterior hippocampus: DG **(F)**, CA4 **(G)**, CA2/3 **(H)**, CA1 **(I)**, and Sub **(J)**. Columns represent mean oligodendrocyte number, and bars represent standard error of the mean. Light gray columns: left hemisphere; dark gray columns: right hemisphere. No significant differences were found between patients with definite and possible cognitive deficits. Right column **(K–O)**: Number of oligodendrocytes [×10^5^] in SZ with definite and possible cognitive deficits in entire (anterior + posterior) hippocampus: DG, **(K)**, CA4 **(L)**, CA2/3 **(M)**, CA1 **(N)**, and Sub **(O)**. Columns represent mean oligodendrocyte number, and bars represent standard error of the mean. Light gray columns: left hemisphere; dark gray columns: right hemisphere. Decreased oligodendrocyte numbers in SZ with definite cognitive deficits were observed in DG (left and right) and CA2/3 (left). *p < 0.05.

**Figure 3:**
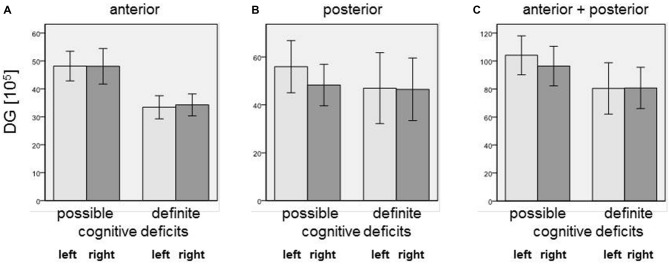
**Number of neurons [×10^5^] in (A) anterior, (B) posterior, and (C) entire DG in SZ patients with definite and possible cognitive deficits.** Columns represent means, and bars represent standard error of the mean.

Our simple classification of cognitive deficits indicated that patients with a definite cognitive dysfunction had a more pronounced reduction of oligodendrocyte total cell numbers (combined staining with cresyl violet (Nissl) and myelin (luxol fast blue), Schmitt et al., 2009; Falkai et al., 2016) than patients with only a possible cognitive deficit. In the anterior hippocampus, SZ patients with definite cognitive deficits showed significantly lower numbers of oligodendrocytes in the left CA1 (−40%, *p* = 0.027), left CA2/3 (−40%, *p* = 0.050), left (−45%, *p* = 0.014) and right (−25%, *p* = 0.050) CA4, and left (−48%, *p* = 0.050) and right (−52%, *p* = 0.014) subiculum. In the posterior part of the hippocampus, definite cognitive deficits appeared to have no significant influence, while in the entire hippocampus SZ patients with definite cognitive deficits showed decreased oligodendrocyte numbers in the left (−42%, *p* = 0.050) and right (−41%, *p* = 0.050) DG and left CA2/3 (−35%, *p* = 0.050; Figure 2). In patients with definite cognitive dysfunction, the number of neurons was not significantly lower in the DG of the anterior, posterior, or entire hippocampus (Figure 3). However, it has to be noted that patients with definite cognitive deficits had received a higher daily dose of neuroleptics during the last 90 days expressed in CPE compared to patients with only a possible cognitive deficit (1906 ± 1333 vs. 388 ± 302 mg/day, *p* = 0.032).

### Densities of Olig1-, Olig2- and Parvalbumin-Positive Cells

**Figure 4:**
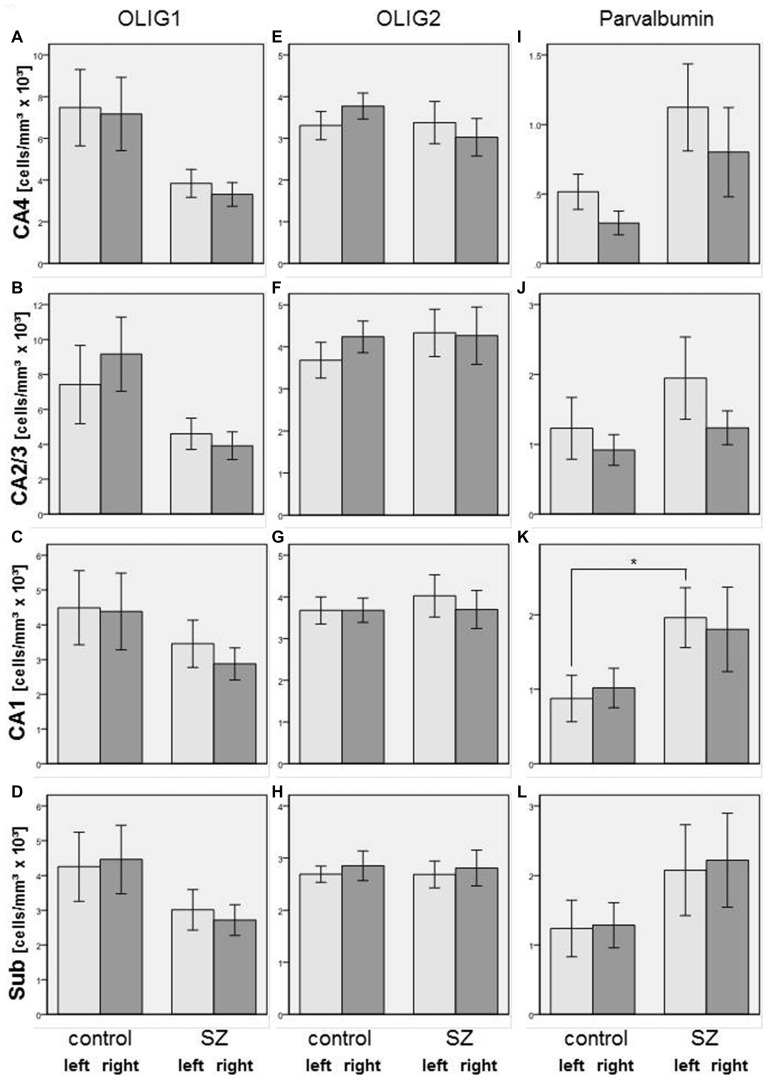
**Left column (A–D): Cell density (cells/mm^3^ × 10^3^) in CA4 (A), CA2/3 (B), CA1 (C), and subiculum (Sub, [D]) of Olig1-positive oligodendrocytes in SZ patients and controls.** Columns represent mean cell density, and bars represent standard error of the mean. Light gray columns: left hemisphere; dark gray columns: right hemisphere. Middle column **(E–H)**: Cell density (cells/mm^3^ × 10^3^) in CA4 **(E)**, CA2/3 **(F)**, CA1 **(G)**, and Sub **(H)** of Olig2-positive oligodendrocytes in SZ and controls. Columns represent mean cell density, and bars represent standard error of the mean. Light gray columns: left hemisphere; dark gray columns: right hemisphere. Right column **(I–L)**: Cell density (cells/mm^3^ × 10^3^) in CA4 **(I)**, CA2/3 **(J)**, CA1 **(K)**, and Sub **(L)** of parvalbumin-positive interneurons in SZ and controls. Columns represent mean cell density, and bars represent standard error of the mean. Light gray columns: left hemisphere; dark gray columns: right hemisphere. Increased density of parvalbumin-stained cells in SZ compared to controls was observed in left CA1. *p < 0.05.

We did not find significant differences between SZ and controls in any of the subregions of the posterior hippocampus in the number of Olig1-, Olig2-, or parvalbumin-positive cells. However, in the SZ we found trends towards a decrease in Olig1-positive oligodendrocytes in the right CA2/3 (−57%, *p* = 0.070), right CA4 (−54%, *p* = 0.096), and right subiculum (−39%, *p* = 0.082). The SZ and controls showed no significant differences with respect to the mean densities of Olig2-positive oligodendrocytes in the investigated subregions of the posterior part of the hippocampus, but the density of parvalbumin-stained interneurons was increased in the left CA1 region in the SZ (+124%, *p* = 0.033) and showed a trend towards an increase in the right CA4 (+175%, *p* = 0.067; see Figure 4).

**Figure 5:**
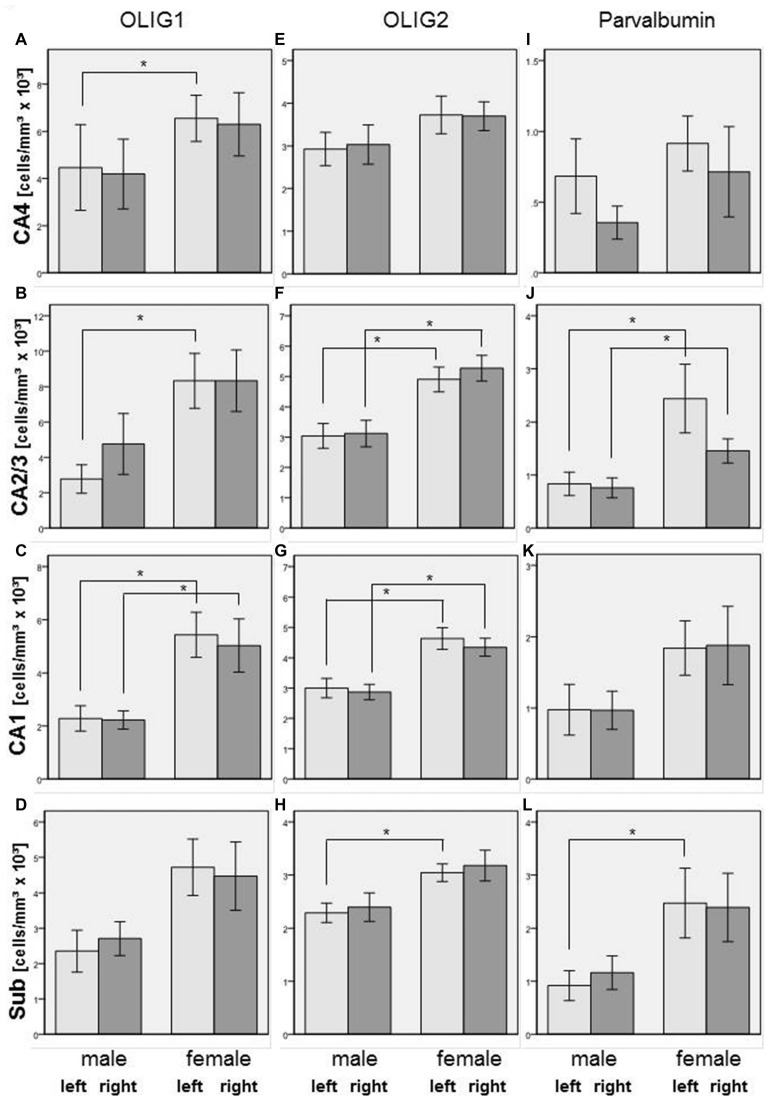
**Left column (A–D): Cell density (cells/mm^3^ × 10^3^) in CA4 (A), CA2/3 (B), CA1 (C), and subiculum (Sub, [D]) of Olig1-positive oligodendrocytes in male and female subjects in the posterior hippocampus.** Columns represent mean cell density, and bars represent standard error of the mean. Light gray columns: left hemisphere; dark gray columns: right hemisphere. Increased Olig1-positive oligodendrocytes in females compared to males were observed in left and right CA1, in left CA2/3 and in left CA4. *p < 0.05. Middle column **(E–H)**: Cell density (cells/mm^3^ × 10^3^) in CA4 **(E)**, CA2/3 **(F)**, CA1 **(G)**, and subiculum **(H)** of Olig2-positive oligodendrocytes in males and females in the posterior hippocampus. Columns represent mean cell density, and bars represent standard error of the mean. Light gray columns: left hemisphere; dark gray columns: right hemisphere. Increased density of Olig2-positive oligodendrocytes in females compared to males were observed in left and right CA1, left and right CA2/3 and in left subiculum (Sub) *p < 0.05. Right column **(I–L)**: Cell density (cells/mm^3^ × 10^3^) in CA4 **(I)**, CA2/3 **(J)**, CA1 **(K)**, and subiculum **(L)** of parvalbumin-positive interneurons in males and females in the posterior hippocampus. Columns represent mean cell density, and bars represent standard error of the mean. Light gray columns: left hemisphere; dark gray columns: right hemisphere. Increased density of parvalbumin-stained cells in females compared to males has been detected in left and right CA2/3 and in left subiculum (Sub) *p < 0.05.

In the total sample of SZ and healthy controls, females showed higher Olig1 cell densities than males in the left (+138%, *p* = 0.009) and right (+126%, *p* = 0.005) CA1, left CA2/3 (+201%, *p* = 0.008), and left CA4 (+47%, *p* = 0.026) subregions. With respect to Olig2-stained cells, females had increased cell densities in the left (+54%, *p* = 0.011) and right (+52%, *p* = 0.004) CA1, left (+61%, *p* = 0.007) and right (+69%, *p* = 0.006) CA2/3, and left subiculum (+33%, *p* = 0.007). Furthermore, females had an increased density of parvalbumin-positive interneurons compared to males in the left (+192%, *p* = 0.016) and right (+92%, *p* = 0.032) CA2/3, and left subiculum (+169%, *p* = 0.033; see Figure 5).

**Figure 6:**
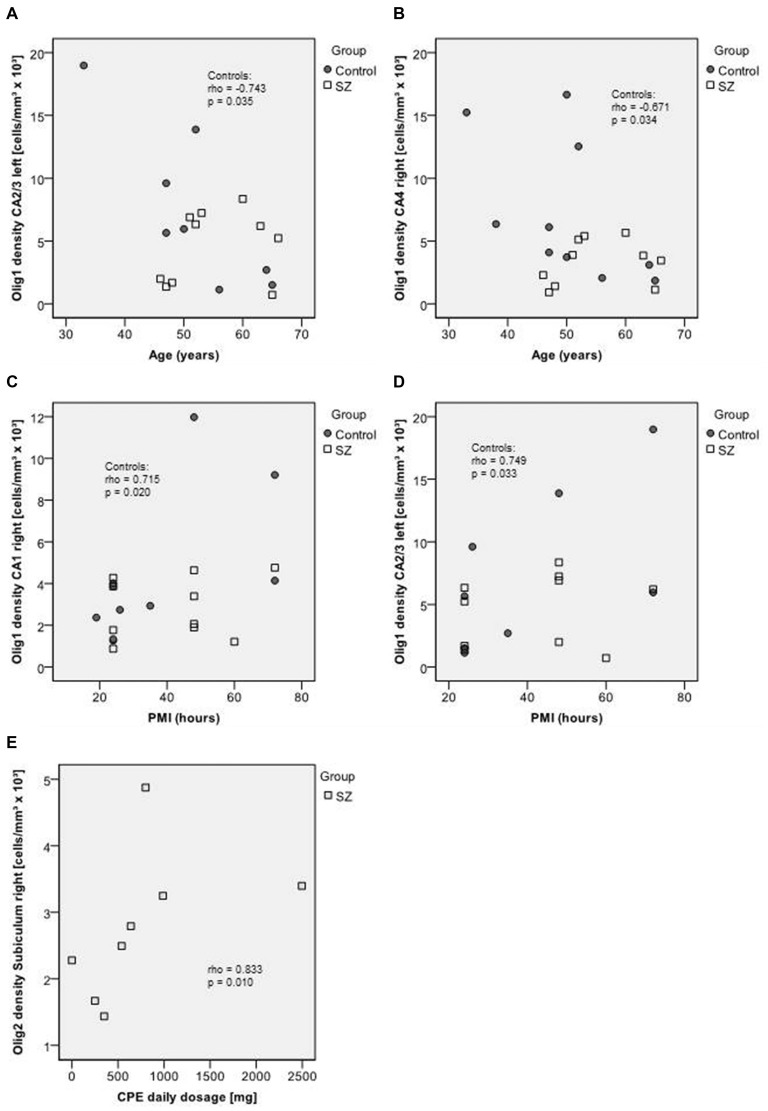
**Upper section (A–B): scatterplots showing the relation between age and Olig1 densities in CA2/3 left (A) and in CA4 right (B).** Middle section **(C–D)**: scatterplots showing the relation between post mortem interval (PMI) and Olig1 densities in CA1 right **(C)** and CA2/3 left **(D)**. Bottom **(E)**: scatterplot showing the relation between chlorpromazine equivalents (CPE) and Olig2 density in the right subiculum. Control subjects appear as dark circles, SZ appear as light squares.

In healthy controls, age correlated negatively with the density of Olig1-positive cells in the left CA2/3 (rho = −0.743, *p* = 0.035) and right CA4 (rho = −0.671, *p* = 0.034), whereas in SZ no significant correlations between age and cell densities were observed. In controls, the PMI correlated with oligodendrocyte density (Olig1, CA1 right: rho = 0.715, *p* = 0.020, CA2/3 left: rho = 0.749, *p* = 0.033), but not with Olig2 or interneuron density. No significant correlations of Olig1-, Olig2- or interneuron densities with disease duration were observed. The Olig2 density correlated with CPE in the right subiculum (rho = 0.833, *p* = 0.010), but no other correlations with dose of neuroleptic treatment had been found (Figure 6).

Furthermore, the densities of Olig1-, Olig2-, and parvalbumin-stained cells did not differ between patients with definite and possible cognitive deficits in any of these areas of the posterior hippocampus.

## Discussion

Our recent design-based stereology investigation of the posterior part of the hippocampus in post-mortem schizophrenia brains revealed a significant bilateral reduction of oligodendrocytes in the left and right CA4 regions (Schmitt et al., 2009). In a subsequent study, we evaluated the anterior part of the hippocampus in the same cases and calculated results for the entire hippocampus. This study replicated the reduction of oligodendrocyte numbers, but only for the left CA4 region in schizophrenia (Falkai et al., 2016). Noteworthy is that no other histological components, including neurons and astroglia, showed alterations in the anterior or posterior portion of the hippocampus in schizophrenia, which is in contrast to the reduction of interneurons in schizophrenia found in a stereological study of the hippocampus and published also in reviews (Konradi et al., 2011; Heckers and Konradi, 2015). However, the study by Konradi et al. (2011) provided stereological data. Also, our evaluation of cell densities may have more methodological bias based on effects of tissue shrinkage which is caused by fixation or staining procedures.

To connect the reduction of oligodendrocytes with clinical outcome, we used the available case notes to subdivide the SZ into those with definite or possible cognitive dysfunction. Nine of the 10 patients showed either definite or possible deficits. Of interest is that on the basis of our stereological data we found a relation between definite cognitive dysfunction and reduced oligodendrocytes in several subregions of the anterior and entire hippocampus, which might point to a central role of oligodendrocytes in cognitive dysfunction in schizophrenia. Oligodendrocytes are important in myelination and are an integral part of the connectome, which allows brain regions to communicate with each other efficiently and quickly (Cassoli et al., 2015). Therefore, we hypothesize that a reduction in oligodendrocytes in the hippocampus might well be an underlying psycho-architectural substrate of cognitive dysfunction in schizophrenia. The fornix, which comprises the white matter tract of the hippocampus, shows decreased fractional anisotropy in first-episode and chronic SZ (Rametti et al., 2009; Abdul-Rahman et al., 2011; Kunimatsu et al., 2012; Fitzsimmons et al., 2014), and these alterations in the fornix correlate with impairment in declarative-episodic memory (Kuroki et al., 2006; Lim et al., 2006). Moreover, oligodendrocyte-related gene variants are related to white matter tract integrity and cognitive performance in SZ and healthy controls (Voineskos et al., 2013). Our results and those findings from magnetic resonance imaging studies support the hypothesis of structural and functional disconnectivity in a hippocampal-medial prefrontal network, leading to profound cognitive deficits in schizophrenia (Hutcheson et al., 2015).

Because in our stereological studies (Schmitt et al., 2009; Falkai et al., 2016), Nissl staining allows oligodendrocytes to be distinguished only morphologically from interneurons, we used Olig1, Olig2, and parvalbumin to stain sections adjacent to those in the same brains previously evaluated by design-based stereology. In accordance with our findings of unchanged neuron numbers in the Nissl-staining stereological study, we found no difference between schizophrenia and healthy controls in the density of a subgroup of 20% of interneurons stained by parvalbumin in the posterior part of the hippocampus (Freund and Buzsaki, 1996). This is in contrast to previously reported reductions of the total number of parvalbumin-positive interneurons in CA4 and CA1 and the density of parvalbumin-stained interneurons in CA2 (Benes et al., 1998; Knable et al., 2004) or all subfields (Zhang and Reynolds, 2002). However, these studies have conflicting results with respect to the affected subregions, and they did not separate the anterior from the posterior part of the hippocampus. Moreover, two-dimensional counting of cell density in only a few sections without considering the volume of the region has methodological limitations because of the influence of volume differences and tissue shrinkage resulting from fixation and staining procedures. Bias may be caused also by cutting of cells during sectioning, non-random orientation, and irregular cell shape and size (Williams and Rakic, 1988). Therefore, design-based stereological studies of the posterior part of the hippocampus are necessary to elucidate the impact of decreased inhibition by a marked deficit of subclasses of interneurons.

Although the reduction of Olig1-positive oligodendrocytes in CA4 did not reach significance, the trend towards significant reduction is in the same direction as in our stereological study and partly supports the notion of reduced oligodendrocyte numbers in the posterior part of the hippocampus in schizophrenia (Schmitt et al., 2009; Falkai et al., 2016). Oligodendrocytes stained by Olig1 represent myelinating and oligodendrocyte progenitors (Arnett et al., 2004), and immunolabeling with other oligodendrocyte markers such as 2’,3’-cyclic nucleotide 3’-phosphodiesterase (CNP), GalC or myelin basic protein (MBP) is necessary to investigate possible myelin damage or whether more mature forms of these glia cells are altered in schizophrenia.

A limitation of our study is that it was only possible to identify cognitive deficits by evaluating medical records, and no structured neuropsychological *in vivo* test results were available. However, the rater (J. Steiner) is an experienced psychiatrist, and case notes were detailed, because of the long-term hospitalization of the patients. In addition, the sample size was small and, because we did not perform Bonferroni correction, results should be replicated in an independent sample. Prospective post-mortem studies that use standardized tests to investigate cognitive deficits in brain donors before death also are warranted to confirm our results. A further limitation is the long-term treatment with typical neuroleptics, which may have influenced our results. The patient group with definite cognitive deficits had received higher doses compared to patients with only possible cognitive deficits. However, from clinical practice it is known that patients with persisting symptoms receive higher doses of antipsychotics than patients in remission. Additionally, neuroleptic treatment may have influenced cell densities in our study. Haloperidol is known to increase expression of Olig2 in the hippocampus and cerebral cortex, whereas quetiapine and olanzapine increase expression of both Olig1 and Olig2 (Wang et al., 2010; Fang et al., 2013). Additionally, haloperidol and clozapine have been shown to be protective for energy-deprived immature oligodendrocytes (Steiner et al., 2011). Moreover, after cuprizione-induced demyelination, quetiapine enhanced oligodendrocyte regeneration and myelin repair (Zhang et al., 2012). Thus, the unchanged density of Olig1- and Olig2-positive cells in the SZ in our study may be a consequence of increased expression of oligodendrocytes due to long-term antipsychotic treatment, which counteracted the decreased expression of oligodendrocytes previously reported in these patients (Schmitt et al., 2009; Falkai et al., 2016). Moreover, the high variance in the data for the SZ may be due to treatment with different neuroleptics. However, because some variances in density were significantly higher in controls than in SZ, e.g., Olig1 densities in CA2/3 and CA4 (compare Figure 4) it would be speculative to explain this high variance by the type of antipsychotic medication and dosage. In addition, the density of parvalbumin-positive interneurons may also have been affected by treatment, because risperidone has been found to normalize loss of this cell population after prenatal immune activation in rats (Piontkewitz et al., 2012). However, in healthy animals haloperidol and clozapine did not change the density of parvalbumin-stained interneurons (Cahir et al., 2005). Finally, age may have influenced our results, because we found a negative correlation between age and Olig1 density in CA4 and CA2/3; however, this correlation was found only in healthy controls and not in SZ. We detected a clear gender effect on cell densities, but this is irrelevant for our group comparisons because there were equal numbers of males and females in both the schizophrenia and control groups.

In summary, we could show that a loss of oligodendrocytes in the anterior and entire hippocampus is related to cognitive deficits in this patient group. However, we did not find significant differences in Olig1-, Olig2-, or parvalbumin-positive cell densities in any subregions of the posterior hippocampus in SZ compared to healthy controls. Here, we did find a trend towards a reduction of Olig1-stained cells in CA4, which points in the same direction as the decrease of oligodendrocyte number in this area in schizophrenia found in our previous stereological studies (Schmitt et al., 2009; Falkai et al., 2016). Further studies investigating white matter tract integrity of the posterior hippocampus and its relationship to cognitive deficits and oligodendrocyte gene variants are needed to further illustrate the impact of oligodendrocyte dysfunction on cognition in schizophrenia. Furthermore, animal models of gene-environment interaction may elucidate the relationship between oligodendrocyte loss and cognitive deficits.

## Author Contributions

JSt, JSh, AK, TS-A, TK, H-GB, AH, BB, PF, BM and AS were involved in data acquisition, statistical evaluation, and manuscript preparation. PF, BM, JSt, AS, and BB contributed substantially to the conception design and interpretation of the work. All authors drafted and finally approved the work and agreed for publication.

## Funding

The study was supported by the European Commission under the Sixth Framework Programme (BrainNet Europe II, LSHM-CT-2004–503039). Furthermore, this work was funded by the German Federal Ministry of Education and Research (BMBF) through the Integrated Network IntegraMent (Integrated Understanding of Causes and Mechanisms in Mental Disorders) under the auspices of the e:Med Programme (grant number 01ZX1314I to PF and TK). Additionally, work was funded by the EU project IN-SENS FP7-PEOPLE-2013-ITN (607616). The article reflects only the views of the authors and the Community is not liable for any use that may be made of it.

## Conflict of Interest Statement

BM, JSh, AK, TS-A, TK, JSt, H-GB, and BB declare no conflicts of interest. AH has been invited to scientific meetings by Lundbeck, Janssen-Cilag, and Pfizer, and he received a paid speakership from Desitin, Otsuka, and Lundbeck and was member of an advisory board of Roche and Lundbeck. PF has been an honorary speaker for AstraZeneca, Bristol Myers Squibb, Eli Lilly, Essex, GE Healthcare, GlaxoSmithKline, Janssen Cilag, Lundbeck, Otsuka, Pfizer, Servier, and Takeda. During the past 5 years, but not presently, PF has been a member of the advisory boards of Janssen-Cilag, AstraZeneca, Eli Lilly, and Lundbeck. AS has been an honorary speaker for TAD Pharma and Roche and has been a member of advisory boards for Roche.
